# Suppression of Expression Levels of Constitutive Androstane Receptor by Moderate Exercise in BALB/c Nude Mice with Breast Cancer

**Published:** 2017-08

**Authors:** Bang-Sub LEE, Wi-Young SO, Wooyoung CHUNG, Eun-Ju CHOI

**Affiliations:** 1. Sports and Health Care Major, Korea National University of Transportation, Chungju-Si, Korea; 2. Dept. of College of General Education, Chung-Ang University, Seoul, Korea; 3. Division of Sport Sciences, Konkuk University, Chungju-Si, Korea

## Dear Editor-in-Chief

The effect of exercise in increasing survival rates from breast cancer is mainly from improvements in maximal oxygen consumption (VO_2_max) and a better body composition (1), as various studies have reported benefits in postoperative recovery and success of chemotherapy (2). However, the exact mechanism for these benefits remains unclear.

The role of constitutive androstane receptor (CAR) with respect to efficacy in cancer therapy has been presented in various studies. CAR is a nuclear receptor encoded by the NR1I3 gene in humans (3). CAR plays a pivotal role in detoxification and clearance of toxic substances, including anti-cancer drugs (3). Besides, CAR induces the activation of cytochromes P450, a regulator of xenobiotics metabolism in liver (3). In addition, the inhibition of CAR-mediated pathway has been suggested as likely to improve the effectiveness of chemotherapy in ovarian cancer cells, an observation explained by increasing the sensitivity of anticancer agents and overcoming drug resistance in these cells (4). The same study showed that treatment with thiazole-5-cabaldehyde O- (3,4-dichlorobenzyl) oxime (CITCO), a CAR specific agonist, led to an impairment of anti-cancer agent action as well as inhibition of cancer cell apoptosis (4). As such, the effectiveness of anticancer agent depended on the metabolism or clearance of CAR by the liver.

To the best of our knowledge, a link between breast cancer therapy, CAR levels and regular exercise was not suggested in that study or elsewhere. Finding such linkage could benefit the approach to treating the breast cancer patients. To this end, we hypothesized that the exercise on regular basis in a mice model with induced breast cancer would reduce the expression of CAR in the liver and improve anti-cancer treatment in test animals, translating to an accelerated postoperative recovery in patients. The design of the study was to investigate the effects of treadmill running for 16-weeks on the expression of CAR mRNA in liver of mice with breast cancer.

All experiments were conducted in accordance with the National Institutes of Health guidelines for care and use of animals. Fifteen female BALB/C nude mice were purchased through Charles River Japan (6 weeks, 20.0 ± 0.5g, Charles River Inc. Japan.). Mice were randomly assigned to a control (non-cancer group; NG, n = 5), a cancer group (CG, n = 5), and a cancer group with exercise (CEG, n = 5). The CEG performed treadmill exercise with the exercise protocol (5). The intensity of this treadmill protocol was 70% to 75% of murine maximal oxygen uptake (6). NG and CG did not have an exercise regimen.

For cancer model, the MCF-7 human breast cancer cell line was used in the nude mice (ATCC; Manassas, VA, USA). The protocol for the cancer model is described in Salvo et al. (7) CAR expression was detected using the real-time PCR in each independent group. Primer sequences for CAR mRNA detection were ggaggaccagatctcccttc for the forward primer and atttcattgccactcccaag for the reverse primer. All values were calculated as mean ± standard error of mean. Group differences in the mRNA of CAR were determined by one-way analysis of variance with the Least Significant Difference post-hoc test using SPSS version 18.0 (SPSS, Chicago, IL, USA). The significance level was set at *P* < 0.05.

**Fig. 1:**
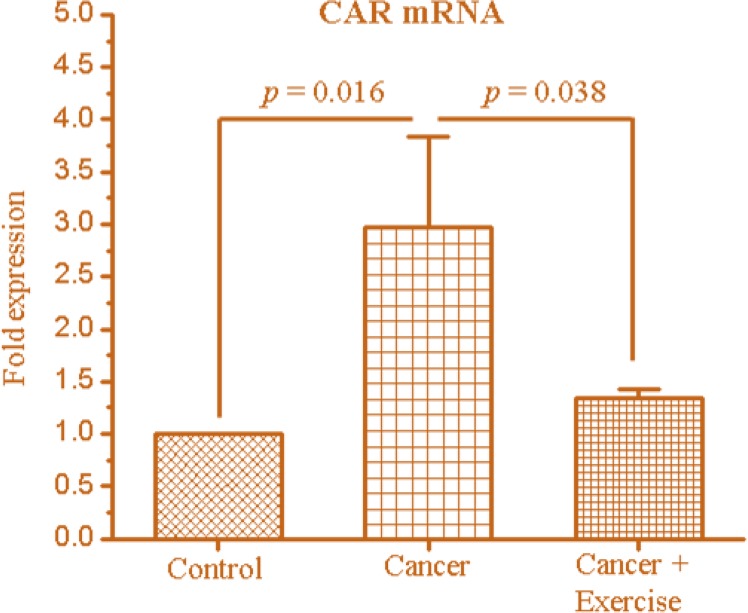
The differences in the expression of the constitutive androstane receptor (CAR) mRNA of liver in MCF7 breast cancer-bearing animals. Fold expression was normalized to non-cancer (control). *P* values were estimated by the Least Significant Difference post-hoc test

We examined the effect of exercise on CAR expression in the liver in breast cancer bearing mice. Fig. 1 shows the expression of CAR in the NG, CG and CEG. The relative fold expression of CAR with relative to NG livers in CG and CEG was 2.97 ± 0.85 and 1.33 ± 0.08, respectively. CAR expression in CG was significantly increased compared with NG (p = 0.016). Moreover, CAR in CEG was significantly lower than the CG (*P* = 0.038), but not compared with the NG.

These results suggest that exercise is likely to inhibit the increases seen in CAR levels in the liver of the cancer subjects, enabling one of the benefits of regular exercise in cancer therapy.

## References

[1] Irwin ML Alvarez-Reeves M Cadmus L (2009). Exercise improves body fat, lean mass, and bone mass in breast cancer survivors. Obesity (Silver Spring), 17: 1534–1541. 19629060 10.1038/oby.2009.18 PMC2841468

[2] Backman M Browall M Sundberg CJ Wengström Y (2016). Experiencing health - Physical activity during adjuvant chemotherapy treatment for women with breast cancer. Eur J Oncol Nurs, 21: 160–7. 26463702 10.1016/j.ejon.2015.09.007

[3] Lake BG Price RJ Osimitz TG (2015). Mode of action analysis for persticide-induced rodent liver tumors involving activation of the constitutive androstane receptor: relevance to human cancer risk. Pest Manag Sci, 71: 829–834. 25045103 10.1002/ps.3854

[4] Wang Y Masuyama H Nobumoto E Zhang G Hiramatsu Y (2014). The inhibition of constitutive androstane receptor-mediated pathway enhances the effects of anticancer agents in ovarian cancer cells. Biochem Pharmacol, 90: 356–366. 24928535 10.1016/j.bcp.2014.06.003

[5] Jones LW Eves ND Courneya KS Chiu BK Baracos VE Hanson J Johnson L Mackey JR (2005). Effects of exercise training on anti-tumor efficacy of doxorubicin in MDA-MB-231 breast cancer xenografts. Clin Cancer Res, 11: 6695–6698. 16166449 10.1158/1078-0432.CCR-05-0844

[6] Fernando P Bonen A Hoffman-Goetz L (1993). Predicting submaximal oxygen consumption during treadmill running in mice. Can J Physiol Pharmacol, 71: 854–857. 8143245 10.1139/y93-128

[7] Li Z Carrier L Belame A Thiyagarajah A Salvo VA Burow ME Rowan BG (2009). Combination of methylselenocysteine with tamoxifen inhibits MCF-7 breast cancer xenografts in nude mice through elevated apoptosis and reduced angiogenesis. Breast Cancer Res Treat, 118: 33–43. 18855134 10.1007/s10549-008-0216-x

